# Blood glucose to predict symptomatic intracranial hemorrhage after endovascular treatment of acute ischemic stroke with large infarct core: a prospective observational study

**DOI:** 10.3389/fneur.2024.1367177

**Published:** 2024-05-01

**Authors:** Yujie Yang, Lihui Yang, Xiaolei Shi, Xuan Ni, Shitao Fan, Xu Xu, Jinfu Ma, Shihai Yang, Zhixi Wang, Wenjie Zi, Dahong Yang, Yonggang Hao

**Affiliations:** ^1^Department of Neurology, The Fourth Affiliated Hospital of Soochow University, Suzhou, China; ^2^Department of Neurology, Xinqiao Hospital and the Second Affiliated Hospital, Army Medical University (Third Military Medical University), Chongqing, China; ^3^Department of Pharmacy, The Fourth Affiliated Hospital of Soochow University, Suzhou, China

**Keywords:** large infarct core, symptomatic intracranial hemorrhage, endovascular treatment, acute ischemic stroke, glucose

## Abstract

**Introduction:**

Symptomatic intracranial hemorrhage (sICH) is a serious complication of acute ischemic stroke (AIS) after endovascular treatment (EVT). Limited data exist regarding predictors and clinical implications of sICH after EVT, underscoring the significance of identifying risk factors to enhance prevention strategies. Therefore, the main objective of this study was to evaluate the incidence of sICH and identify its predictors after EVT in patients with large infarct core-AIS in the pre-circulation stage.

**Methods:**

Using data from the EVT for the Pre-circulation Large Infarct Core-AIS Study, we enrolled patients who were treated with EVT from the Prospective Multicenter Cohort Study of Early Treatment in Acute Stroke (MAGIC) registry. Baseline demographics, medical history, vascular risk factors, blood pressure, stroke severity, radiographic features, and EVT details were collected. The patients were classified into three groups: without intracranial hemorrhage (ICH), with asymptomatic intracranial hemorrhage (aICH), and sICH, based upon the occurrence of sICH. The main outcomes were the occurrence of sICH according to the Heidelberg Bleeding Classification and functional condition at 90 days. Multivariate logistic regression analysis and receiver operating characteristic (ROC) curves were used to identify independent predictors of sICH after EVT.

**Results:**

The study recruited a total of 490 patients, of whom 13.3% (*n* = 65) developed sICH. Patients with sICH had less favorable outcomes than those without intracranial hemorrhage (ICH) and those with aICH (13.8% vs. 43.5% vs. 32.2%, respectively; *p* < 0.001). The overall mortality was 41.8% (*n* = 205) at 90 days post-EVT. The univariate analysis revealed significant differences among the three groups in terms of blood glucose levels at admission, probability of favorable outcomes, incidence of brain herniation, and 90-day mortality. The multifactorial logistic regression analysis revealed that the blood glucose level at admission [odds ratio (OR) 1.169, *p* < 0.001, confidence interval (CI) 1.076–1.269] was an independent predictor of sICH. A blood glucose level of 6.95 mmol/L at admission was the best predictor of sICH, with an area under the ROC curve (AUC) of 0.685 (95% CI: 0.616–0.754).

**Discussion:**

The study findings demonstrated that the probability of sICH after EVT was 13.3% in patients with pre-circulation large infarct core-AIS, and sICH increased the risk of an unfavorable prognosis. Higher blood glucose levels at admission were associated with sICH after EVT in patients with pre-circulation large infarct core AIS. These findings underscore the importance of early management strategies to mitigate this risk.

## 1 Introduction

As the second most lethal and disabling disease worldwide, acute ischemic stroke (AIS) has a serious impact on patient survival and quality of life (1). Approximately 20% of patients with AIS arrive at the hospital with a large infarct core, characterized by high mortality and poor prognosis, posing a global challenge in clinical management for this population (2). Endovascular therapy (EVT) has emerged as the preferred treatment option for AIS due to large vessel occlusion.

A previous study reported that, compared with patients treated with medication alone, those with large infarct-core AIS treated with EVT plus medication had a higher likelihood of reduced disability, independent ambulation, and favorable functional outcomes at 90 days (3). The Chinese Guidelines for Endovascular Treatment of Acute Ischemic Stroke 2023 recommend EVT for patients with large-vessel occlusion-induced AIS in the pre-circulation stage among those who meet the enrolment criteria of the ANGEL-ASPECT (4), RESCUE-Japan LIMIT (5), or SELECT 2 (6) studies (7).

Despite the success associated with EVT, intracranial hemorrhage (ICH) is a common complication, with a prevalence of 46.0–49.5% (8). In particular, symptomatic ICH (sICH) can reduce the benefit–risk ratio of treatment and increase the risk of mortality (9). However, limited data are available regarding the predictors and clinical relevance of sICH after EVT. Therefore, risk factors for sICH after EVT should be identified for the prevention of sICH and improvement in the efficacy of this new treatment strategy. Utilizing the database from the Prospective Multicenter Cohort Study of Early Treatment in Acute Stroke (MAGIC) study, the present study aimed to analyze potential predictors of sICH after EVT in patients with pre-circulation large infarct-core AIS.

## 2 Materials and methods

### 2.1 Patients

Patients were enrolled from the database of the MAGIC study, a nationwide prospective registry of consecutive patients who presented with acute, symptomatic, and radiologically confirmed pre-circulation large infarct core AIS. The MAGIC registry included patients with AIS with a large infarct core due to pre-circulation large-vessel occlusion who underwent EVT from November 2021 to February 2023. This prospective observational study was approved by the ethics committees of the participating centers.

The eligibility criteria for EVT were as follows: (1) age 18–80 years; (2) AIS due to anterior circulation large vessel occlusion, defined as occlusion of the internal carotid artery (ICA) or the M1 segment or M2 segment of the middle cerebral artery (MCA); (3) large ischemic core on non-contrast computed tomography (CT) findings [defined as Alberta stroke programme early CT score (ASPECTS) score of 0–5]; (4) pre-stroke score of 0 or 1 on the modified Rankin scale (mRS), assessed retrospectively (scores ranging from 0 to 6, with higher scores indicating greater disability and a score of 6 indicating death); and (5) symptom presentation within 24 h (the time metric of time last known well within 24 h was used if the presentation time was unavailable). The exclusion criteria were as follows: (1) no follow-up brain imaging data (CT or magnetic resonance imaging) at 24 h or when neurological deterioration occurred; (2) serious, advanced, or terminal illness; and (3) no mRS data at 90 days.

### 2.2 Clinical data collection

The collected data included baseline demographic data (age and sex), medical history, vascular risk factors (smoking, hypertension, hyperlipidemia, diabetes, and atrial fibrillation), blood pressure at admission (systolic and diastolic), stroke severity [National Institutes of Health Stroke Score (NIHSS)], radiographic features (ASPECTS, site of the occluded arteries), and EVT-related data (procedure process time, treatment methods, and recanalization). Additional data analyzed included blood glucose level at admission, triglyceride level, low-density cholesterol level, liver function, kidney function, and intravenous thrombolytic therapy (IVT).

The site of the occluded arteries was identified using CT angiography (CTA), magnetic resonance angiography (MRA), and/or cerebral digital subtraction angiography (DSA) reports and included the ICA and MCA. Anterior circulation lesions were defined using the ASPECTS score, which involved symptom onset to puncture (OTP) time and symptom onset to recanalization (OTR) time.

The patients in our study received EVT, which included intra-arterial thrombolysis, thrombectomy with stent retrievers, thromboaspiration, intracranial angioplasty, stent implantation, or a combination of these approaches at the discretion of the treatment surgeon.

### 2.3 Post-procedure evaluation

CT was usually performed 24 h after the procedure or whenever ICH was indicated by clinical symptoms. Successful recanalization was defined as a modified treatment in cerebral infarction (mTICI) with a score of 2b, 2c, or 3. The mRS score was evaluated at 90 days by a stroke neurologist during a scheduled post-stroke follow-up visit or via phone interview. Functional outcomes were evaluated according to the mRS as follows: complete recovery (mRS = 0–1), partial recovery, independence (mRS = 2), dependence (mRS = 3–5), and death (mRS = 6). Favorable and unfavorable outcomes were defined as an mRS score of 0–3 and > 3 (4–6), respectively.

### 2.4 Evaluation of ICH

According to the Heidelberg Bleeding Classification, ICH is diagnosed within 48 h after EVT. sICH and asymptomatic intracranial hemorrhage (aICH) were classified based on the presence or absence of neurological deficit exacerbations. The diagnosis of sICH was based on the association of ICH with any of the following conditions: (1) increase in NIHSS score by >4 points compared to the score prior to ICH; (2) increase in NIHSS score by >2 points in one category; and (3) deterioration leading to intubation, hemicraniectomy, external ventricular drain placement, or any other major intervention. Symptom deteriorations were required to be unexplainable by causes other than the observed ICH (10).

### 2.5 Statistical analysis

Measures with normal distribution are expressed as mean ± standard deviation of variance (ANOVA) with Bonferroni correction used for multiple comparisons. Continuous variables are presented as medians [interquartile range (IQR)] according to the type of non-normal distribution, and categorical variables are presented as frequencies (percentages). The Wilcoxon rank-sum test was used for comparison between two groups, and the Kruskal–Wallis test was used for multiple comparisons. Categorical variables were analyzed using the chi-squared test or Fisher’s exact test. A multivariate logistic regression analysis was performed to evaluate independent predictors for sICH, with the adjusted odds ratio (OR) and corresponding 95% confidence interval (CI) reported. Receiver operating characteristic (ROC) curve analysis was used to evaluate the optimal cutoff value for predicting sICH and to establish optimal cutoff points for the specificity and sensitivity values. Entered factors were those with at least marginal significance (*p* < 0.1) in a univariate analysis. *p*-values of <0.05 were considered significant. Statistical analyses were performed using SPSS 23.0. (IBM, Armonk, NY, United States).

## 3 Results

**Figure 1:**
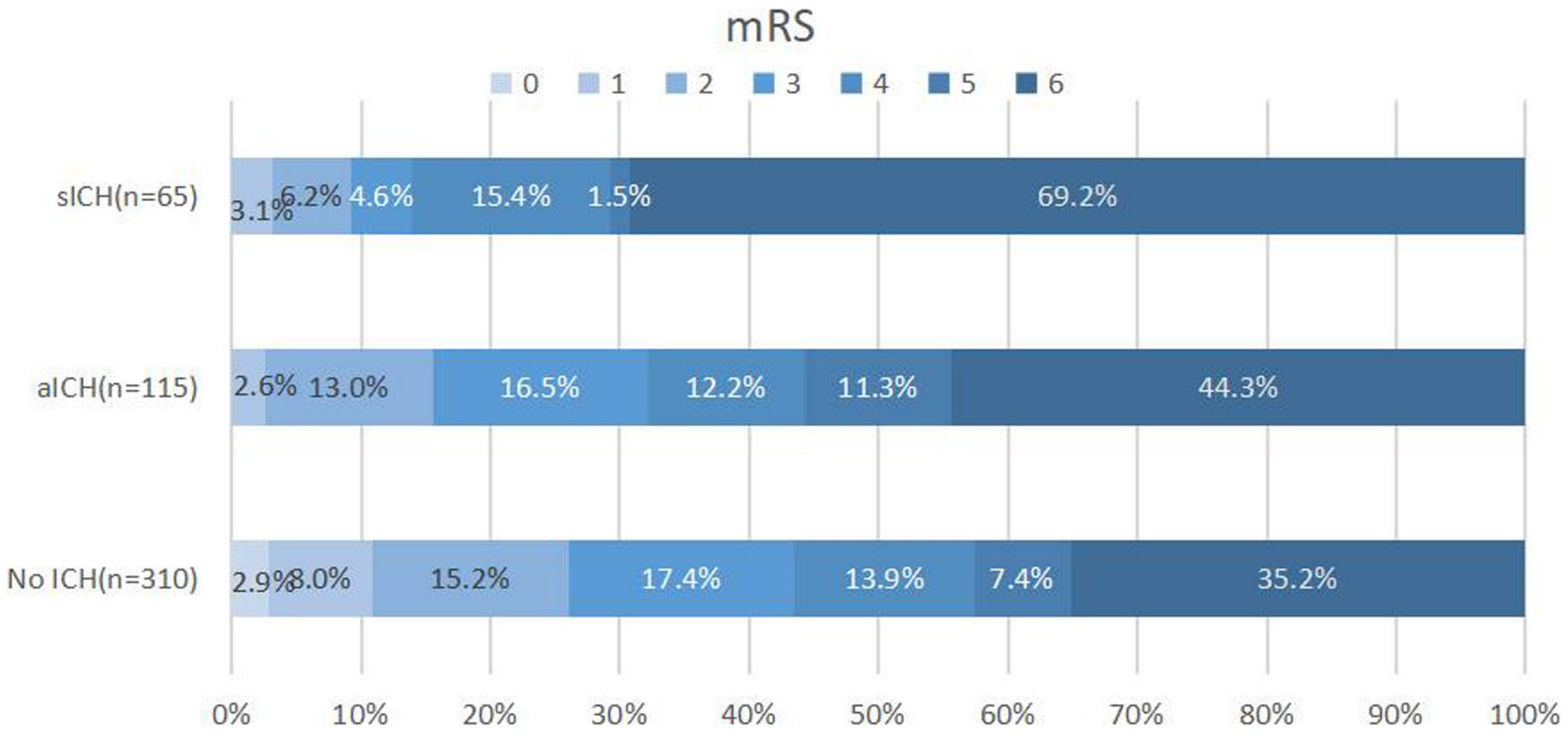
Distribution of 90-day mRS scores in patients without ICH, aICH, and sICH. Patients with sICH demonstrated the least favorable outcomes (mRS 0–3) 90 days post-index stroke, with a higher proportion experiencing mortality. The distribution of modified Rankin scale (mRS) scores underscores the impact of sICH on long-term prognosis. mRS, modified Rankin scale; ICH: intracranial hemorrhage; aICH, asymptomatic intracranial hemorrhage; sICH, symptomatic intracranial hemorrhage.

A total of 490 patients with pre-circulation large infarct-core AIS who were treated with EVT were enrolled in this study. They were divided into three groups: without ICH, with aICH, and with sICH. Overall, 115 patients (23.5%) had aICH after EVT within 24 h, whereas 65 patients (13.3%) had sICH. The proportions of reaching favorable functional outcomes in the three groups were 43.5, 32.2, and 13.8%, respectively (*p* < 0.001) (Figure 1 and Table 1). Significant differences were noted in the probability of developing brain herniation (21.6% vs. 30.4% vs. 58.5%, *p* < 0.001) and mortality at 90 days (35.2% vs. 44.3% vs. 69.2%, *p* < 0.001) (Table 1).

**Table 1:** Baseline characteristics and outcomes of patients without ICH, with aICH, and sICH.

Variables	ALL (*n* = 490)	No sICH		sICH (*n* = 65)	*P*-value
		Without ICH (*n* = 310)	aICH (*n* = 115)		
Male gender, *n* (%)	281 (57.3)	178 (57.4)	73 (63.5)	30 (46.2)	0.078
Age (years)	68 ± 12	68 ± 12	68 ± 12	68 ± 11	0.911
AF, *n* (%)	221 (45.10%)	137 (44.2%)	52 (45.2%)	32 (49.2%)	0.759
Hypertension, *n* (%)	297 (60.6%)	197 (63.5%)	67 (58.3%)	33 (50.8%)	0.134
DM, *n* (%)	73 (14.9%)	46 (14.8%)	14 (12.2%)	13 (20.0%)	0.366
Hyperlipidemia, *n* (%)	106 (21.6%)	68 (21.9%)	28 (24.3%)	10 (15.4%)	0.365
Smoke, *n* (%)	151 (30.8%)	101 (32.6%)	37 (32.2%)	13 (20.0%)	0.128
SBP (mmHg)	146.7 ± 25.7	146.9 ± 24.8	146.6 ± 26.8	145.9 ± 28.5	0.969
DBP (mmHg)	86.2 ± 15.8	86.2 ± 15.1	85.2 ± 14.9	88.1 ± 20.3	0.527
Glucose (mmol/L)	7.9 ± 2.9	7.6 ± 2.8	8.0 ± 2.9	9.25 ± 3.5	<0.001
Triglyceride, (mmol/L) (median, IQR)	1.2 (0.8–2.2)	1.3 (0.8–2.2)	1.2 (0.9–2.2)	1.2 (0.7–2.2)	0.784
Cholesterol (mmol/L)	4.0 ± 1.4	4.0 ± 1.4	3.9 ± 1.4	4.2 ± 1.4	0.560
LDL-C (mmol/L)	2.5 ± 0.9	2.5 ± 0.9	2.4 ± 0.9	2.5 ± 0.9	0.790
Creatinine (umol/L) (median, IQR)	72.2 (61.0–89.6)	72.1 (60.0–91.0)	73.20 (64.0–92.1)	65.3 (57.20–81.3)	0.335
BUN (mmol/L) (median, IQR)	5.8 (4.6–7.5)	5.7 (4.6–7.6)	5.8 (4.6–7.6)	5.7 (4.6–7.3)	0.943
Baseline measurements					
ASPECTS median (IQR)	4 (2–5)	4 (2–5)	4 (2–5)	3 (2–5)	0.214
Baseline_NIHSS median (IQR)	17 (14–20)	17 (13–20)	16 (13–20)	17 (13–21)	0.137
Occlusion site, *n* (%)					0.110
ICA	206 (42.0%)	121 (39.0%)	49 (42.6%)	36 (55.4%)	
M1	233 (47.6%)	157 (50.6%)	55 (47.8%)	21 (32.3%)	
M2	51 (10.4%)	32 (10.3%)	11 (9.6%)	8 (12.3%)	
Procedure process and results					
Intravenous thrombolysis, *n* (%)	122 (24.9%)	78 (25.2%)	32 (27.8%)	55 (84.6%)	0.667
Local anesthesia, *n* (%)	405 (82.7%)	258 (83.2%)	92(80.0%)	46 (88.5%)	0.221
OTP, min, median (IQR)	362 (240–47)	360 (235–532)	360 (240–593)	439 (299–521)	0.153
OTR, min, median (IQR)	450 (326–657)	427 (320–640)	475 (330–680)	503 (359–633)	0.088
mTICI 2b, 2c, 3, *n* (%)	423 (86.3%)	266 (85.8%)	102 (88.7%)	55 (84.6%)	0.677
Cerebral hernia, n(%)	140 (28.6%)	67 (21.6%)	35 (30.4%)	38 (58.5%)	<0.001
90-d mRS ≤3, *n* (%)	181 (36.9%)	135 (43.5%)	37 (32.2%)	9 (13.8%)	<0.001
90-d Mortality, *n* (%)	205 (41.8%)	109 (35.2%)	51 (44.3%)	45 (69.2%)	<0.001

Data are presented as mean and standard deviation unless mentioned otherwise.

ICH, intracranial hemorrhage; sICH, symptomatic intracranial hemorrhage; aICH, asymptomatic intracranial hemorrhage; IQR, interquartile range; DM, diabetic mellitus; AF, atrial fibrillation; SBP, systolic blood pressure; DBP, diastolic blood pressure; LDL-C, low-density lipoprotein-cholesterol; BUN, blood urea nitrogen; ASPECTS, Alberta Stroke Program Early Computed Tomography Score; NIHSS, National Institute of Health Stroke Scale; ICA, internal carotid; M1, middle cerebral artery proximal; M2, middle cerebral artery distal; OTP, symptoms onset to puncture time; OTR, symptoms onset to recanalization time; mTICI, modified thrombolysis in cerebral infarction; mRS, modified Rankin scale.

**Figure 2:**
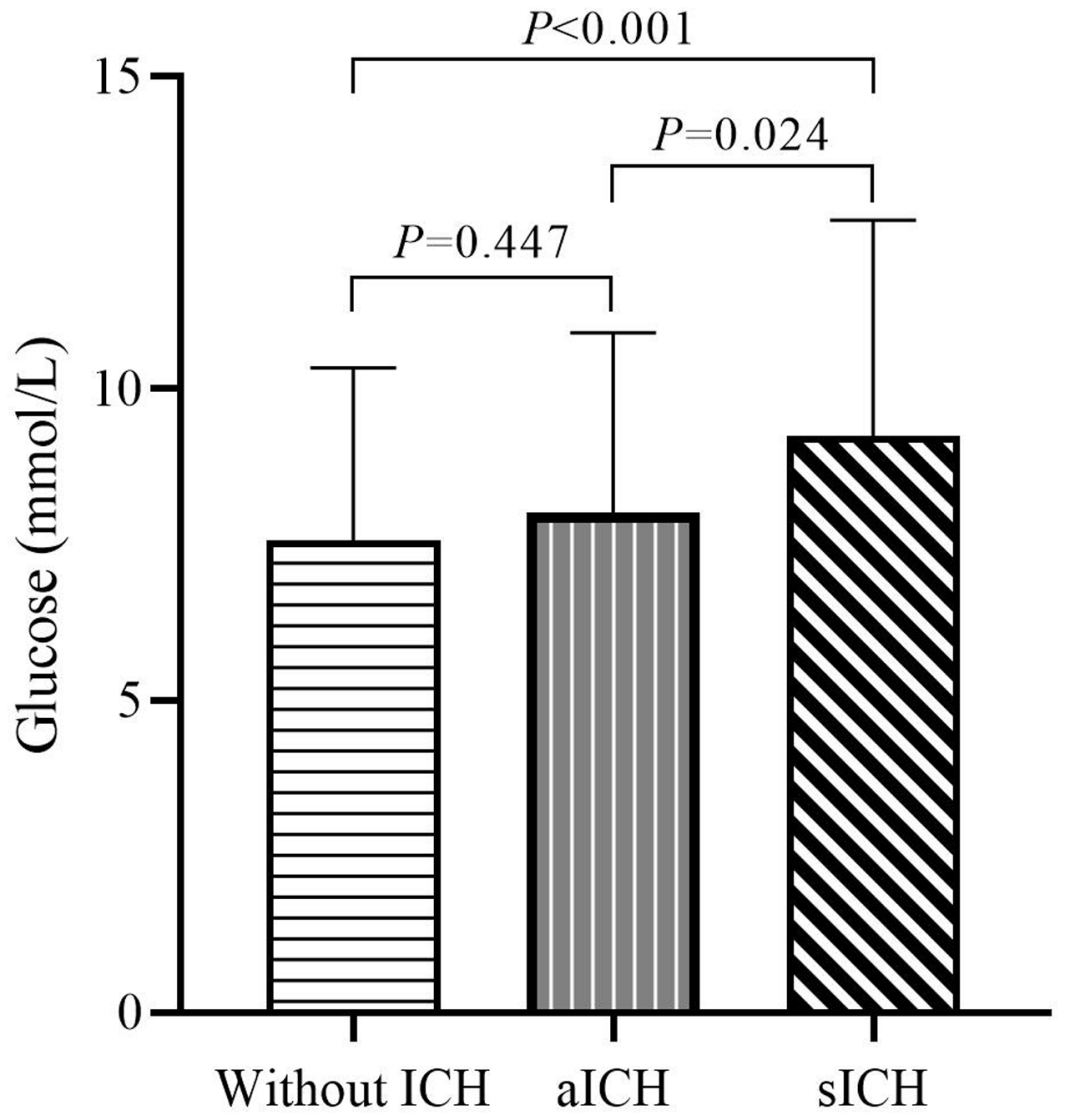
Admission glucose levels in patients without ICH, aICH, and sICH. Significant differences in admission glucose levels were observed between groups without ICH, aICH, and sICH. The sICH group exhibited elevated blood glucose levels compared to the other two groups, emphasizing the association between admission hyperglycemia and symptomatic intracranial hemorrhage (after the Bonferroni correction).

When comparing sICH based on the level of blood glucose at admission, a significant intergroup difference was noted (7.6 ± 2.8 vs. 8.0 ± 2.9 vs. 9.25 ± 3.5 mmol/L, respectively, *p* < 0.001) (Table 1). The one-way ANOVA revealed that the levels of blood glucose at admission in the sICH group were significantly different from those in the without ICH and with aICH groups (*p* < 0.001; *p* = 0.008, respectively). The count showed that the levels of blood glucose at admission in the sICH group were significantly different from those in the without ICH and with aICH groups (p < 0.001; *p* = 0.024, respectively, by the Bonferroni correction) (Figure 2). In multivariate analysis, higher blood glucose levels at admission (OR 1.169, p < 0.001, CI 1.076–1.269) were associated with sICH after EVT in patients with pre-circulation large infarct-core AIS (Table 2).

**Table 2:** Multiple multifactorial logistic regression predicts sICH after EVT.

Variable	B	OR	95% CI		P
			Lower	Upper	
Male gender	0.279	1.322	0.746	2.341	0.339
Glucose level	0.156	1.169	1.076	1.269	0.000*
OTR	0.000	1.000	0.999	1.001	0.406

sICH, symptomatic intracranial hemorrhage; EVT, endovascular treatment; OTR, symptom onset to recanalization time.

**Figure 3:**
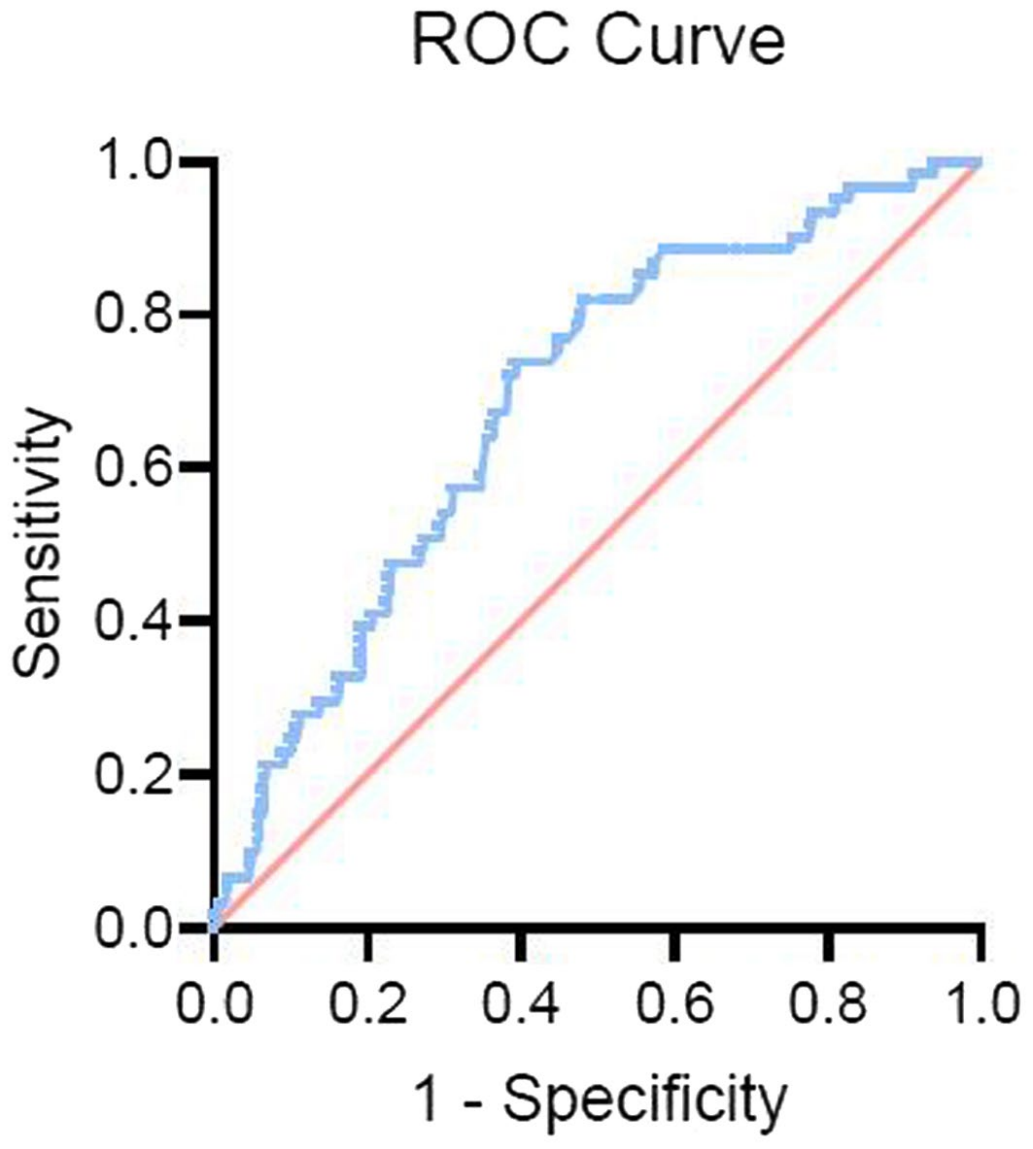
Receiver operating characteristic (ROC) curve for blood glucose levels predicting sICH. The ROC curve illustrates the predictive value of admission blood glucose levels for symptomatic intracranial hemorrhage (sICH). The curve highlights the discriminative ability of blood glucose levels, with an optimal threshold identified for predicting the occurrence of sICH after endovascular therapy (EVT).

Figure 3 presents the results of the ROC analysis determining the prognostic value of blood glucose levels at admission to predict sICH. The area under the curve for the model was 0.685, with a 95% CI of 0.616–0.754, indicating that blood glucose levels at admission have a good discriminative ability. The optimal threshold was 6.95 mmol/L, corresponding to a sensitivity and specificity of 82.0 and 49.8%, respectively, with Youden’s index of 0.312. The positive predictive value was 19.2%, and the negative predictive value was 84.1%.

## 4 Discussion

In our study, we explored potential predictors of sICH after EVT in patients with pre-circulation large infarct-core AIS and observed that the probability of sICH was 13.3% in patients who underwent EVT. Furthermore, sICH greatly reduced the probability of patients having a 90-day favorable prognosis and increased the risk of brain herniation and mortality. In addition, we demonstrated that the blood glucose level at admission was an independent predictor of sICH after EVT in AIS patients with large infarct cores, and the risk of sICH increased with higher blood glucose levels.

In our study, the occurrence of sICH was similar to that in the SELECT trial, which reported an increased probability of sICH in patients with large-vessel occlusion of large-core infarcts who were treated with EVT with an onset greater than 24 h (11). However, our results showed a higher value (13.3%) than that reported in a previous randomized controlled trial (4.4%) (12) and in the North American Solitaire Acute Stroke Study (9.9%) (13). This discrepancy may stem from the inclusion of patients in our study with large infarct cores in the anterior circulation and an ASPECTS score of ≤5, whereas previous EVT studies have largely excluded patients who had large infarct cores already present at the time of preoperative imaging. Several studies have demonstrated an association between larger cores on imaging and an increased risk of sICH after EVT (14, 15). In most studies, an ASPECTS score of ≤5 was reported to be associated with an approximately 2-fold risk of ICH (16, 17). Patients with large infarct cores have a high degree of blood–brain barrier disruption, and the development of ICH is closely related to blood–brain barrier damage. In previous models of cerebral ischemia in rats, loss of integrity of the matrix and basement membrane connecting the endothelial cells was observed by electron microscopy, and in some cases, glial cell protrusions were observed to be swollen or degenerative. These alterations affect the structure and function of the blood–brain barrier, which induces ICH by increasing its permeability (18).

Experimental results have shown that the ischemia–reperfusion-induced release and activation of metalloproteinases can cause rupture of the basement membrane, leading to ICH (19, 20). The results of a previous meta-analysis demonstrated that, compared to pharmacological treatment, EVT did not increase the risk of sICH but improved the functional outcome in patients with large infarct cores (21, 22). Based on the above observations, it is reasonable to attribute the higher incidence of sICH in our study to the larger infarct core rather than the EVT procedure itself.

Similar to the findings of Shen et al. (23), our results showed that patients with sICH had a higher risk of brain herniation and mortality and a greater likelihood of unfavorable outcomes than patients without ICH and with aICH. This emphasizes the critical nature of sICH as a potentially fatal complication that significantly influences patient functional outcomes, warranting emphasis on preventive measures.

In our study, blood glucose was identified as a predictor of sICH after EVT in AIS patients with large infarct cores in the anterior circulation. The blood glucose levels >6.95 mmol/L at admission were associated with an increased risk of sICH after EVT, comparable to a level of 6.6 [5.7–7.7] mmol/L reported in a previous study (24). Several previous studies have suggested that patients with hyperglycemia are at a high risk of ICH from different perspectives, such as a meta-analysis that retrospectively analyzed the clinical data of patients undergoing EVT and found that higher blood glucose levels at admission were associated with a higher incidence of sICH (25, 26). Previous studies have shown that acute hyperglycemia increases blood–brain barrier disruption and ICH incidence in rat models (27). The results of Shen et al.’s (23) study suggest that high blood glucose levels are the strongest predictors of sICH after EVT. Another study showed that an elevated glycosylated hemoglobin level was significantly associated with an unfavorable prognosis and mortality in 90 days with AIS treated with EVT (28). The results of these studies are consistent with our findings; however, the mechanisms underlying how hyperglycemia enhances ICH remain unclear. The possible causes of ICH are as follows: (1) hyperglycemia exacerbates vascular wall dystrophy and hypoxia, resulting in more susceptible degeneration and necrosis of the vascular wall (29, 30); (2) hyperglycemia leads to disruption of cellular metabolism, resulting in increased plasma osmolality and intracellular lactic acid buildup, ultimately leading to endothelial cell injury and acidosis (31); and (3) hyperglycemia can reportedly increase the activity of the matrix metalloproteinases (MMP-9 and MMP-3) in ischemic areas and aggravate blood–brain barrier dysfunction and post-reperfusion ICH (32–34). While it is hypothesized that maintaining stable blood glucose levels post-successful EVT may be crucial in preventing sICH (35), further studies are needed to confirm this.

This study has some limitations that should be considered when interpreting the results. First, as the study adopted a prospective observational design, we did not evaluate other potentially relevant variables, such as changes in perioperative blood pressure, use of heparin during EVT, and the effect of antiplatelet regimens on hemorrhagic conversion, which may influence the risk of ICH. Second, an incomplete evaluation index may not fully determine a causal connection, and the practical implications of these unresolved findings require future validation. Despite these limitations, the findings of this study provide insights into the predictive value of blood glucose levels at admission for sICH after EVT in pre-circulation large infarct core-AIS to facilitate early management.

## 5 Conclusion

In this multicenter study, the incidence of sICH after EVT was higher than that previously reported. Patients with pre-circulation large infarct core AIS who developed sICH after EVT had a higher incidence of unfavorable prognosis and mortality than those who did not develop sICH. Higher blood glucose levels at admission increased the risk of sICH after EVT in AIS patients with large infarct cores in the anterior circulation. It remains unclear whether controlling blood glucose levels prior to EVT can reduce the risk of sICH, and further investigation is required.

## Data availability statement

The original contributions presented in the study are included in the article/supplementary material, further inquiries can be directed to the corresponding author.

## Ethics statement

The studies involving humans were approved by the ethics committee of Suzhou Dushu Lake Hospital. The studies were conducted in accordance with the local legislation and institutional requirements. The participants provided their written informed consent to participate in this study.

## Author contributions

YY: Writing – original draft, Data curation, Formal analysis, Investigation. LY: Writing – original draft, Data curation, Methodology. XS: Writing – original draft, Formal analysis, Investigation, Software. XN: Formal analysis, Writing – original draft, Data curation, Funding acquisition, Methodology. SF: Data curation, Writing – original draft, Conceptualization, Investigation, Supervision. XX: Investigation, Supervision, Data curation, Writing – original draft, Formal analysis, Methodology. JM: Methodology, Data curation, Writing – original draft, Software, Validation. SY: Methodology, Writing – original draft, Conceptualization, Project administration, Resources, Supervision. ZW: Methodology, Writing – original draft, Formal analysis, Software, Validation. WZ: Validation, Writing – review & editing, Data curation, Funding acquisition, Project administration, Resources. DY: Funding acquisition, Project administration, Resources, Writing – review & editing, Data curation, Investigation, Formal analysis. YH: Investigation, Project administration, Writing – review & editing, Supervision, Visualization.
