# Corrigendum: Blood glucose to predict symptomatic intracranial hemorrhage after endovascular treatment of acute ischemic stroke with large infarct core: a prospective observational study

**DOI:** 10.3389/fneur.2024.1449604

**Published:** 2024-07-05

**Authors:** Yujie Yang, Lihui Yang, Xiaolei Shi, Xuan Ni, Shitao Fan, Xu Xu, Jinfu Ma, Shihai Yang, Zhixi Wang, Wenjie Zi, Dahong Yang, Yonggang Hao

**Affiliations:** ^1^Department of Neurology, The Fourth Affiliated Hospital of Soochow University, Suzhou, China; ^2^Department of Neurology, Xinqiao Hospital and the Second Affiliated Hospital, Army Medical University (Third Military Medical University), Chongqing, China; ^3^Department of Pharmacy, The Fourth Affiliated Hospital of Soochow University, Suzhou, China

**Keywords:** large infarct core, symptomatic intracranial hemorrhage, endovascular treatment, acute ischemic stroke, glucose

In the published article, there was an error. The time to diagnose ICH after EVT was written as 24 h, however the correct time for diagnosing ICH after EVT is 48 h.

A correction has been made to **2. Materials and methods**, *2.4 Evaluation of ICH*, Paragraph 1. This sentence previously stated:

“According to the Heidelberg Bleeding Classification, ICH is diagnosed within 24 h after EVT.”

The corrected sentence appears below:

“According to the Heidelberg Bleeding Classification, ICH is diagnosed within 48 h after EVT.”

The authors apologize for this error and state that this does not change the scientific conclusions of the article in any way. The original article has been updated.

